# Determination of Bitterness of *Andrographis Herba* Based on Electronic Tongue Technology and Discovery of the Key Compounds of Bitter Substances

**DOI:** 10.3390/molecules23123362

**Published:** 2018-12-19

**Authors:** Xiao Zhang, Hongwei Wu, Xiankuo Yu, Hanyan Luo, Yaqi Lu, Hongjun Yang, Xin Li, Zhiyong Li, Liying Tang, Zhuju Wang

**Affiliations:** 1 Institute of Chinese Materia Medica, China Academy of Chinese Medical Science, No.16 Nanxiaojie, Dongzhimennei Ave., Beijing 100700, China; zhangxiaoyx163@163.com (X.Z.); whw9905012@163.com (H.W.); yuxiankuobz@126.com (X.Y.); lhanyan7@163.com (H.L.); luyaqi412@163.com (Y.L.); hjyang@icmm.ac.cn (H.Y.); 18406557239@163.com (X.L.); 2 College of Pharmacy, Henan University of Chinese Medicine, No.156 Jinshuidong Ave., Zhengzhou 450046, China; 3 State Key Laboratory of Innovative Natural Medicine and TCM Injections, Jiangxi Qingfeng Pharmaceutical Co. Ltd., No. 8 Zhandongdong Ave., Ganzhou 341000, China; jxlzy2@qfyy.com.cn

**Keywords:** *Andrographis Herba*, electronic tongue, chromatographic fingerprints, Principal Component Analysis, Spearman correlation analysis, bitter substances

## Abstract

*Andrographis Herba* (AH), the dry aerial segments of *Andrographis paniculata* (Burm.f.) Nees, is a common herbal remedy with bitter properties in traditional Chinese medicine (TCM) theory. Although bitterness is one of the features representing Chinese medicine, it has not been implemented as an index to assess the quality and efficacy of TCM because of peoples’ subjectivity to taste. In this study, 30 batches of AH with different commercial classifications (leaves, stems, or mixtures of both) were collected. Bitterness of AH was quantified by electronic tongue technology. Meanwhile, chemical compositions were characterized through establishing high-performance liquid chromatography fingerprints. The result indicated that the radar curves of the bitterness from different AH commercial classifications displayed different taste fingerprint information. Based on six taste factors, a Principal Component Analysis (PCA) score three-dimensional (3D) plot exhibited a clear grouping trend (R^2^X, 0.912; Q^2^, 0.763) among the three different commercial classifications. Six compounds (Peaks 2, 3, 4, 6, 7, 8) with positive correlation to bitterness were discovered by a Spearman correlation analysis. Peaks 2, 6, 7, 8 were identified as andrographolide, neoandrographolide, 14-deoxyandrographolide, and dehydroandrographolide, respectively. The electronic tongue can be used to distinguish AH samples with different commercial classifications and for quality evaluation.

## 1. Introduction

*Andrographis Herba* (AH, Chuan Xinlian in Chinese) is derived from the dry aerial segments of *Andrographis paniculata* (Burm.f.) Nees, and it has been used in folk medicines for the treatment of fever, common colds, diabetes, hepatitis, skin infections, snake bites, hypertension, and other diseases in several Asian countries, including China, India, and Thailand [1]. Modern pharmacological research has revealed that AH has multiple properties, including anti-inflammatory, bacteriostasis, antioxidative, antitumor, hypoglycemic, cardiovascular, and hepatoprotective [2]. The major active components of AH are considered to be labdane diterpenoid lactones and flavones [3]. In the Pharmacopoeia of the Republic of China, the medicinal part of AH was the aerial segments [4]. However, in fact there are three types of AH samples with different specifications available in the Chinese raw herbal medicine market, which are leaves, stems, or a mixture of both.

According to Traditional Chinese Medicine (TCM) theory, the properties and actions of Chinese herbs refer to their nature and effects relating to treatments. These properties include the “five flavors”, which are tastes composed of pungent, sweet, sour, bitter, and salty, which are high-level summary of the clinical efficacy [5]. For example, some herbal medicines with a bitter flavor possess properties for drying or resolving dampness, purging, and lowering, and are often used to treat constipation due to fired-heat, dysphoria, cough due to the adverse rising of lung-qi, damp heat, or cold damp syndrome [6]. AH is a representative bitter herb, which has the effect of clearing away inner heat, eliminating toxins, drying dampness, and relieving swelling [4]. In the clinic, AH is generally believed that, when compared with stems, AH leaves have better effects. It is subjectively believed that AH leaves are more bitter than stems. Actually, the taste perceived by the mouth is susceptible to physical and psychological conditions, such as differences in the amount of taste buds or personal preferences [7]. Up to now, there were some reports about the quality evaluation of AH samples with different specification by HPLC and UPLC analysis [8,9]. However, the bitterness of AH samples have not been quantitatively and objectively evaluated, and research is lacking on the relationship between the bitterness and chemical compounds of AH.

The use of electronic tongues offers a promising alternative to solve those problems, which consists of an analytical sensor array system that is able to detect specific substances by means of different artificial membranes and electrochemical techniques [10]. Electronic tongues were initially implemented in food sectors in the 1980s [11,12] and later in various other areas, for example, TCM quality control [13,14], taste masked drug formulations [15,16,17,18], and identification of Chinese medicine adulteration [19], as well as for changes in taste before and after TCM processing [20,21].

In this study, electronic tongue technology was firstly used to quantitatively and objectively detect bitterness and evaluate the quality of AH samples with different commercial classifications. Furthermore, a fingerprint method was developed for characterizing chemical composition, and correlating the chemical composition with bitterness to explore the key of bitter compounds of AH.

## 2. Results and Discussion

### 2.1. Bitterness AH Aample Analysis via Electronic Tongue

The electronic tongue divides response range of the “minimum taste stimulation” and the “maximum taste stimulation” into 25 units according to Weber Fechner′s law. Each unit represents that the concentration of the sample is changed by 20%, but if the change lower than this unit, then normal people will not feel the difference between taste stimulation. The range of bitterness and astringency that humans can perceive is 0.00–25.00 [21,22]. The linearity between the concentration of AH samples and sensor responses was observed. The results showed that the sensor responses increased with higher concentrations (0.05–2 g/100 mL). Furthermore, the optimal concentration for determination was 0.1 g samples added to 100 mL of solution, which is detailed in Section 3.3.2. In this concentration, all of the values of the six taste factors were all within the range of 0.00–25.00. The results of repeatability and stability were as follows: precision—the relative standard deviations (RSD) of six taste factors’ values were all less than 5%; and, stability—less than 4%. Thus, all of the results indicated that the electronic tongue measurements were reliable.

Four lipid membrane sensors of bitterness containing six taste factors were used in this study. Table 1 shows the bitterness values of these taste factors in 30 batches of AH samples. There are differences in the six taste factors among AH samples with different commercial classifications, the trends of the changes in these factors are not exactly the same. Thus, the radar curves of bitterness for all AH samples based on the six factors were constructed to comprehensively characterize the taste fingerprint information. 

**Figure 1:**
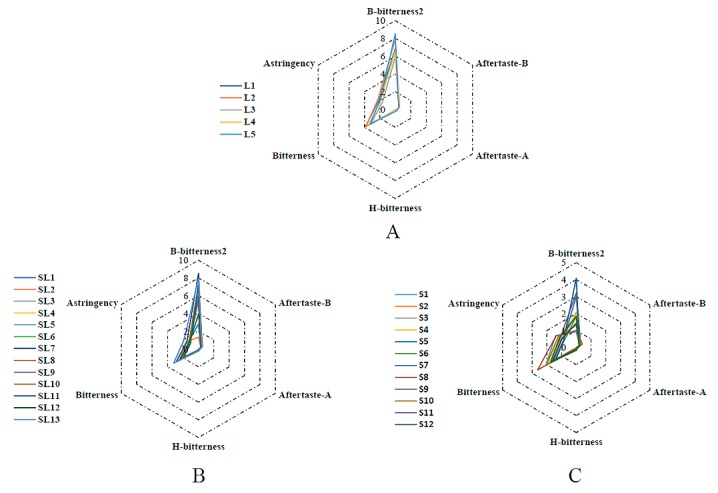
Radar curves of *Andrographis Herba* (AH) samples’ bitterness for different commercial classifications. A: Leaf samples, B: Stem/Leaf samples, C: Stem samples.

As shown in Figure 1, among the six taste factors, B-bitterness2 and Bitterness elicit the strongest responses. The corresponding values of B-bitterness2 range from 0.93 to 8.53, and Bitterness ranges from 1.38 to 3.86 (Table 1). Bitterness as the taste factor of the initial taste represents the taste of medicine in the mouth, whereas B-bitterness2 (i.e., aftertastes of mineral bitterness) represents the taste remaining in the mouth after swallowing. As shown in Table 1, the range and averages of the radar curve areas are as follows: AH leaf samples 7.790–13.449, average of 10.994; stem/leaf samples 2.857–11.067, average of 5.849; stem samples 1.421–2.932, average of 2.034. It can be seen that the bitterness levels are highest in the leaves, followed by stem/leaf combined, and lowest in the stems alone. Therefore, AH samples with different commercial classifications presented different taste fingerprint information, implying that the bitter values, especially the radar curves area, could be used as indexes for the determination of AH samples with commercial classification.

### 2.2. Principal Component Analysis of AH Samples’ Bitterness

**Figure 2:**
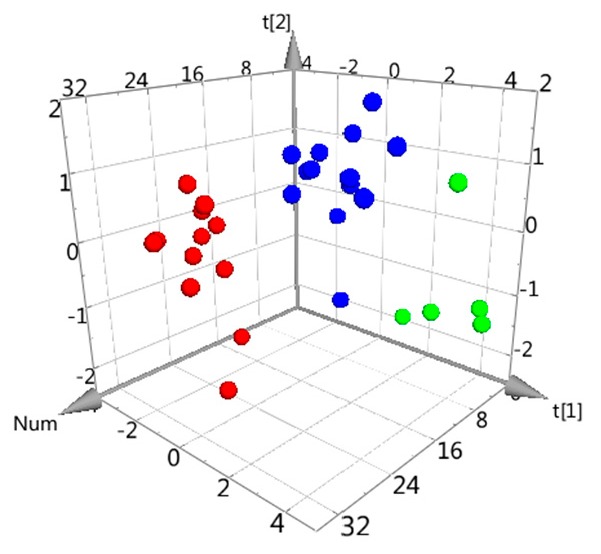
The Principal Component Analysis Three-Dimensional (PCA 3D) scores plot using AH bitterness data. ●: Leaf, ●: Stem/Leaf, ●: Stem.

In order to objectively and visually characterize the differences between AH samples with different commercial classifications, a Principal Component Analysis (PCA) was applied based on the values of the six taste factors. As an unsupervised pattern recognition method, PCA can visualize inherent clustering between different groups, which displays the internal structure of datasets in an unbiased way and decreases data dimensionality [23]. As shown in the PCA score three-dimensional (3D) plot (Figure 2), an overview of all data samples can be observed, which exhibited a clear grouping trend (R^2^X, 0.912; Q^2^, 0.763) among the three classifications of samples. The R^2^X (0.912) and Q^2^ (0.763) represented the PCA model, accounting for 91.2% data variance and a good predictive ability, respectively. This observation indicated that there were indeed differences in bitterness among AH samples with different commercial classifications.

### 2.3. HPLC Fingerprint Analysis

In order to obtain satisfactory efficiency, three extraction methods (refluxing, ultrasonic, and cold-macerating extraction), a range of extraction solvent concentrations (20% methanol, 40% methanol, 60% methanol, 80% methanol, and 100% methanol) and extraction times (0.2 h, 0.3 h, 0.5 h, 1 h) were compared and optimized using univariate tests. The results indicated that there are no obvious differences in the three aforementioned extraction methods. Thus, the most convenient method of ultrasonic extraction was selected. It was found that 40% methanol was the most efficient extraction solvent among the different concentrations based on the main peak areas in the chromatogram. In addition, it was demonstrated that most components could be extracted completely within 0.5 h. In summary, samples were prepared by ultrasonic extraction with 50 mL of 40% methanol for 0.5 h.

**Figure 3:**
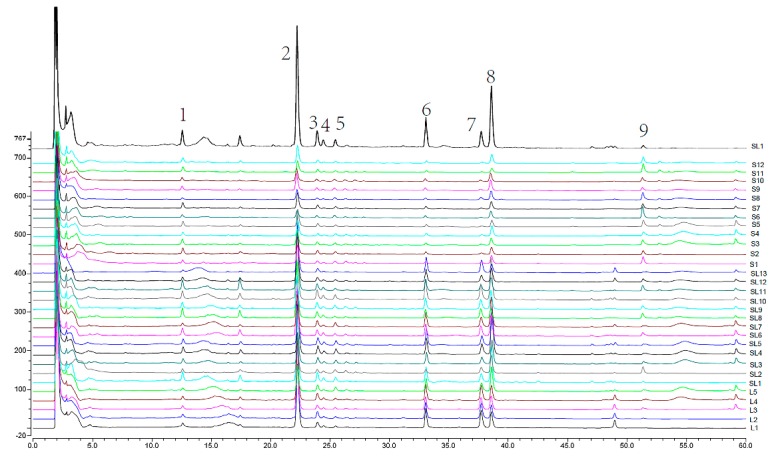
Chromatographic fingerprints of all AH samples. (L1–L5 are leaf samples; SL1–SL13 are stem and leaf mixed samples; S1–S12 are stem samples. Peaks 2,6,7,8 are andrographolide, neoandrographolide, 14-deoxyandrographolide, and dehydroandrographolide, respectively).

As shown in Figure 3, nine common peaks in the chromatogram were selected as the markers for the fingerprints method validation. The relative retention time (RRT) and relative peak area (RPA) of these peaks were calculated for estimation of precision, repeatability, and stability, and the results were as follows: precision—the relative standard deviations (RSD) of RRT and RPA were found not to exceed 0.03% and 2.68%, respectively; repeatability—below 0.07% and 3.14%, respectively; and, stability—less than 0.07% and 3.55%, respectively. Thus, all results indicated that the HPLC measurements were stable and under control.

30 batches of AH samples with different commercial classifications were analyzed, and their corresponding chromatographic fingerprints were aligned and matched using the Similarity Evaluation System for chromatographic fingerprint of TCM (Figure 3). Furthermore, the common peaks of 2, 6, 7, and 8 were identified as andrographolide, neoandrographolide, 14-deoxyandrographolide, and dehydroandrographolide, respectively, by comparison with reference compounds that were based on the ultraviolet spectrum and retention time. With an overview of all samples in the chromatographic fingerprints, the fingerprints’ characteristics vary depending on the commercial classification of the samples. For example, peak 2 in the chromatogram was obviously the highest within the leaf samples, followed by stem and leaf mixed samples, and was lowest in the stem samples. Therefore, in order to discover the key bitter substances from the fingerprint, we further took a correlation analysis between the radar curve areas of the bitter substances and the nine common peak areas of the chromatographic fingerprints.

### 2.4. Spearman Correlation Analysis

**Figure 4:**
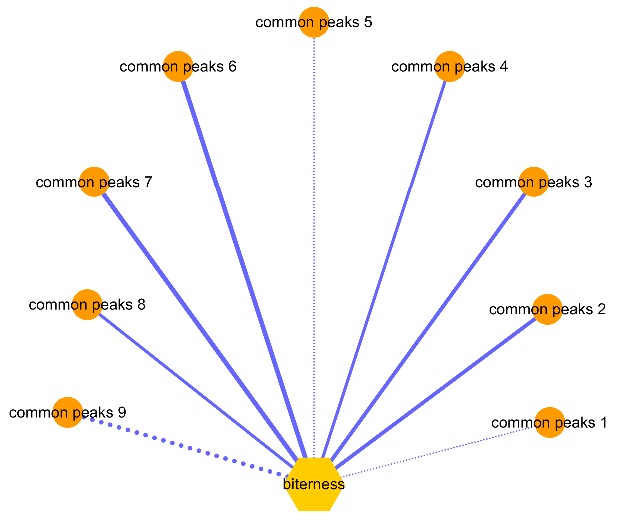
Correlation network between bitterness (radar curve area) and the common peaks in HPLC fingerprint. Visualization of data concentrated on the correlations between chemical constituents in relation to bitterness (radar curve area). The negative correlations are indicated with dots lines, and positive correlations are indicated with solid lines; thicker lines indicate a stronger correlation. The length of each line has no meaning.

In this study, the Spearman correlation analysis was performed to find the key bitter compounds using the Software SPSS21.0 (SPSS Inc., Chicago, IL, USA). The correlation coefficients between bitter (the radar curve area) and common peaks are summarized in Table 2. These correlations are depicted visually in Figure 4. The closer the absolute value of the correlation coefficient is to 1, the more significant the correlation. Generally, if the absolute value of the correlation coefficient was more than 0.5, it indicated a reliable positive or negative correlation (*p* < 0.01).

Of the nine common peaks, the areas of peaks 2, 3, 4, 6, 7, and 8 showed a highly positive correlation with the bitter compounds (the radar curve area), and the corresponding correlation coefficients were 0.725, 0.729 0.629, 0.854, 0.890, and 0.691, respectively. The area of peak 9 showed a highly negative correlation with bitterness, and the correlation coefficient is −0.826. In addition, there were no significant correlations observed between the areas of peaks 1, 5, and the radar curve area of bitter substances. In summary, the more bitter the AH, the higher relative contents of andrographolide (peak 2), neoandrographolide (peak 6), 14-deoxyandrographolide (peak 7), dehydroandrographolide (peak 8), and peaks 3 and 4. In contrast, the more bitter the AH, the lower the relative contents of peak 9.

It was known that AH as a representative TCM with bitter properties is often used for antibacterial and anti-inflammatory remedies in the clinic [24]. The bitter-related substances of andrographolide (peak 2), neoandrographolide (peak 6), 14-deoxyandrographolide (peak 7), and dehydroandrographolide (peak 8) have also been reported with obvious antibacterial and anti-inflammatory effects [3]. Therefore, the bitterness that was detected by the electronic tongue can be used not only to distinguish AH samples with different commercial classifications, but also to reflect the levels of AH effective ingredients. In addition, the results suggested that the other unknown bitter-related substances (Peak 3, 4) may also have bitter activities, such as antibacterial and anti-inflammatory.

## 3. Materials and Methods

### 3.1. Instruments and Chemicals

Bitterness measurements were performed using electronic tongues TS-5000Z (Insent Inc., Atsugi, Japan). HPLC analyses for chromatographic fingerprints were performed with a Dionex U-3000 series (Shanghai, China). HPLC-grade methanol and acetonitrile were supplied by Thermo Fisher Scientific Inc. (Shanghai, China). Deionized water was purchased (Wahaha, China). 20% methanol, 40% methanol, 60% methanol, and 80% methanol were prepared by dilution of absolute methanol with deionized water. The reference compounds andrographolide, dehydroandrographolide, 14-deoxyandrographolide, and neoandrographolide (purity > 95% for all) were purchased from Chengdu Chroma-Biotechnology Co., Ltd. (Sichuan, China). Potassium chloride (analytical grade), tartaric acid (ISO), ethanol (99.8%), hydrochloric acid (36–38%), silver chloride (analytical grade), and potassium hydroxide (ISO) were purchased from Fajiede Chemical Reagent Inc. (Beijing, China).

### 3.2. AH Sample Collection

**Figure 5:**
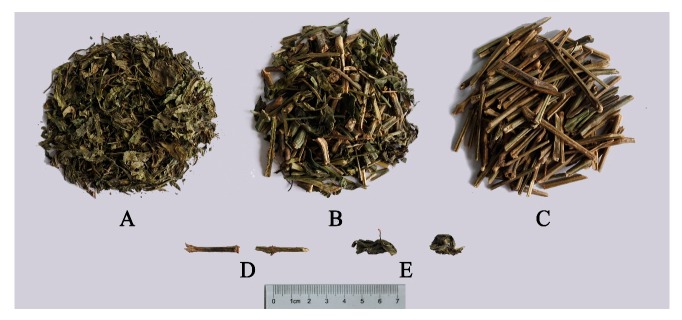
Three typical AH samples with different specification. **A**: leaf sample; **B**: a mixture of leaf and stem sample; **C**: stem sample; **D**: stem; **E**: leaf.

30 batches of AH with different commercial classifications were collected from various provinces in China (Table 3), including their production areas (such as Guangdong, Guangxi, etc.). Samples were denoted, as follows: leaves, L1 to L5; mixed stems and leaves, SL1 to SL13; and, stems, S1 to S12. Three typical AH samples with different specification are shown as Figure 5. In the mixed samples, the ratio of stems to leaves is about 20–50%. The quantity and proportion of three kinds of samples collected represent the actual situation of AH on the market. The samples were identified by Professor Zhuju Wang at the Institute of Chinese Materia Medica, China Academy of Chinese Medical Sciences. All samples were stored in a dry, constant environment to minimize any changes through degradation, and the voucher specimens were deposited in our laboratory.

### 3.3. Electronic Tongue Methods

#### 3.3.1. Electronic Tongue Measurement Principle, Steps, and Conditions 

The measurement principle of electronic tongue TS-5000Z is potentiometric, during measurement, mV values are recorded and no absolute taste values are obtained. The artificial lipid membrane sensor probe is composed of silver-wire electrode, the surface of which is coated with Ag/AgCl, with a sensor body made of polypropylene, and lipid membranes made by mixing lipids (which play an important role in taste sensing) with a polymer [20]. Before each measurement, a sensor check was performed to ensure that the sensors were working in the correct voltage range. During one measurement, every sample was measured four times. For data interpretation, the last three runs were used to enable conditioning of the lipid membranes to the sample solutions and to ensure data stability. 

Every sample measurement starts with a cleaning procedure. After cleaning, the stability of the lipid membrane potential was controlled by measuring the potential of the reference solution (Vr). When the sensor response is stable during 30 s of measurement (deviation smaller than 0.5 mV), the sample solution was measured for 30 s (Vs). Electrical potential changes (R) between Vr and Vs was called the relative potential and used to calculate the initial tastes. After a short cleaning procedure (3 s, two times), the membrane potential is measured again in reference solution for 30 s (Vr’), the change in electric potentials between Vr and Vr’ is called the change of membrane potential that is caused by adsorption (CPA) value and is used to calculate the aftertaste. The CPA value results from the measurement of the adsorption of substances, which are not removed by the short cleaning procedure from the lipid membrane. The electronic tongue detects the membrane potential, and then converts the potential value into a taste value according to Weber Fechner’s law that the intensity of the perception is proportional to the logarithm of stimulus intensity [20].

The electronic tongue TS-5000Z is equipped with up to eight lipid membrane sensors and a reference electrode. The bitter taste is usually represented by three different sensors, labeled C00, BT0, and AN0. In this experiment, except for the three bitterness sensors, an astringency sensor AE1 (auxiliary measurement) was also used to measure bitterness of AH. Especially, the sensors of C00 and AE1 can measure two taste factors, one of which is the initial taste representing the initial taste of medicine in the mouth, whereas the other is the aftertaste representing the taste remaining in the mouth after swallowing (Table 4). Therefore, a total of six values were obtained to characterize the bitterness of the AH samples. The pH range of the sample to be tested should be controlled within 2–8, and all measurement procedures were carried out at the room temperature (23–26 °C).

Potassium chloride and tartaric acid were dissolved in distilled water as reference solution at concentrations of 30 mmol/L and 0.3 mmol/L, respectively, for sensor conditioning and cleaning. For washing the different charged lipid membranes of the sensors, two solutions were prepared: 100 mmol/L hydrochloric acid dissolved in 30% ethanol (made by dilution of absolute ethanol with deionized water) for negatively charged membranes (BT0, AN0); 100 mmol/L potassium chloride and 10 mmol/L potassium hydroxide both dissolved in 30% ethanol for positively charged membranes (AE1, C00). A solution of 3.33 mmol/L potassium chloride in saturated silver chloride was used for sensors and reference electrodes as an inner solution. The sensors were embedded in reference solution for one day prior to being used for measurements.

#### 3.3.2. Sample Preparation for the Electronic Tongue

Each of the dried samples was crushed into a powder with a pulverizer for 2 min and was passed through a 65 μm-mesh sieve. Each sample powder was weighed accurately at 0.1 g, and 100 mL of 10 mmol/L potassium chloride solution was added to increase the conductivity of the solution. After ultrasonication for 30 min, the sample was filtered through gauze and the filtrate was placed in an electronic beaker for testing.

#### 3.3.3. Electronic Tongue Methodology Validation

Methodology validation of electronic tongue was performed for verifying the use of the electronic tongue in AH samples. The linearity was investigated to evaluate the relationship between the sensor responses and the concentrations by measuring the same sample with five different concentrations (0.05 g, 0.1 g, 0.5 g, 1 g and 2 g of AH powders in 100 mL of 10 mmol/L potassium chloride solution). Five parallelly prepared samples from Sample L1 (Table 1) were determined according to the procedure to test repeatability. The sample stability was determined by analyzing a single prepared sample that was stored at room temperature for 0, 1, 2, 4, and 8 h. The RSDs% of the sensor responses were all calculated to estimate repeatability and stability.

### 3.4. HPLC Fingerprint Methods

#### 3.4.1. Chromatographic Conditions

All of the HPLC analyses were performed with a Dionex U-3000 series equipped with a SR-3000 Solvent Rack, a LPG-3400SDN Quaternary Pump, a WPS-3000SL Auto sampler, a TCC-3000RS Column compartment, a DAD-3000RS detector, and a Chromeleon 7 chromatography workstation (Thermo Fisher Scientific, Waltham, MA, USA). An Agilent ZORBAX Extend-C18 column (4.6 mm × 250 mm, 5 μm) was used. The mobile phase consisted of acetonitrile (A) and water (B). The gradient program was developed as follows: 15–28% A for 0–22 min, 28–37% A for 22–35 min, 37–50% A for 35–45 min, and 50–75% A for 45–60 min. The flow rate was maintained at 1.0 mL/min and the column temperature at 30 °C. The injection volume was 10 μL and the detective wavelength was selected at 205 nm.

#### 3.4.2. HPLC Sample Preparation

Each sample powder (0.5 g) was added to a 100 mL Erlenmeyer flask with 50 mL 40% methanol, and the flask was accurately weighed. Following soaking for 1 h and ultrasonic extraction for 30 min, the sample mixture was weighed again and any solvent that was lost in the process was added after being cooled to room temperature. Subsequently, the mixture was filtered through a 0.22 μm membrane filter. Finally, 10 μL aliquots from the filtrate were subjected to HPLC analysis. Stock solutions of the four reference compounds of andrographolide, dehydroandrographolide, 14-deoxyandrographolide and neoandrographolide—of about 0.2 mg/mL—were prepared in methanol and stored at 4 °C for later analysis.

#### 3.4.3. HPLC Methodology Validation

All AH samples were prepared, as described in Section 3.4.2. The precision of the chromatographic method was established by analyzing the same sample solution five times within one day. Precision was expressed as the RSD% of repeated measurements. The sample stability was determined by analyzing a single sample solution that was stored at room temperature for 0, 2, 4, 8, 12, and 24 h. Repeatability was determined by analyzing five separate samples from the same source. The RRT and RPA of each of the common peaks were calculated to estimate precision, stability, and repeatability.

### 3.5. Data Analysis

#### 3.5.1. PCA Analysis for Electronic Tongue Data

The raw data was saved as Common Executable Format (CEF)-files (the rows represent observed samples, the columns represent the variables of bitterness values) and imported into the software of SIMCA-P (Umetrics AB, Umea, Sweden) for PCA employing the Nonlinear Iterative Partial Least Square (NIPALS) algorithm. PCA was performed on the raw data and the pretreatment of the data was performed by UV scaling. PCA, as an unsupervised pattern recognition pattern, generate new original variables, but shows linear combinations of them and simultaneously capture most features of the original data. Thus, PCA decreases the dimensionality of data and could be used to visualize inherent clustering between the AH samples. The score values plots for the first two or three PCs (PC1, PC2, and PC3) are often used to visually represent the characteristics of the samples. The parameters of the modeling, R^2^ and Q^2^ values in PCA, can explain the quality of the fitting model. R^2^ is the percent of variation of the training set—X with PCA—explained by the model. R^2^ is a measure of fit, i.e., how well the model fits the data. Later, R^2^X is the fraction of the variation of the X variables explained by the model. A large R^2^ (close to 1) is a necessary condition for a good model, but it is not sufficient. Q^2^ is the percent of variation of the training set—X with PCA—predicted by the model according to cross validation. Q^2^ indicates how well the model predicts new data. A large Q^2^ (Q^2^ > 0.5) indicates good predictivity [25,26].

#### 3.5.2. Fingerprint Data Processing

The raw HPLC chromatographic data of the 30 tested samples were integrated automatically and exported as *. AIA format files for further processing. Then, all of these files were imported into the Similarity Evaluation System for TCM chromatographic fingerprinting (Version 2004 A; Committee for the Pharmacopoeia of PR China.). One sample was randomly selected as a reference to generate the template. Subsequently, all of the samples were automatically aligned on the basis of this template and the reference peaks. For the chromatograms, which were to be arranged in a line, reference peaks were first aligned to those in the template, and the other peaks were subsequently lined up on the basis of the nearest reference peak in the chromatogram. For further analysis, the retention time and peak area of all aligned peaks were calculated simultaneously and can be exported as an excel file for further statistical analysis.

#### 3.5.3. Correlation Analysis

Input the six taste factors’ value into excel to generate a radar curve, and the area of the radar curve is used to comprehensively represent AH bitterness. The Spearman correlation coefficient is the most commonly used measure of monotone association and it is usually suggested for non-normally distributed data [27]. The data distribution was not normal by the Shapiro–Wilk test and then the Spearman’s rank correlation (*ρ*) was used to quantify the correlation between the radar curve areas and the common peaks in fingerprints (SPSS version 21.0). Significant correlations were defined as Spearman’s |*ρ*| > 0.5 and *p* < 0.01, respectively. Thereafter, Cytoscape version 3.7.0 (www.cytoscape.org) was used to draw a network view to visualize these correlations [28,29].

The Radar curve area was calculated, as follows:

(1) $$\mathrm{Radar}\;\mathrm{curve}\;\mathrm{area}=\sqrt{3}/4\times \left(ab+bc+cd+de+ef+fa\right)$$

where a is B-bitterness2, b is Aftertaste-B, c is Aftertaste-A, d is H-bitterness, and e and f are Bitterness and Astringency, respectively (Table 2).

## 4. Conclusions

In this study, electronic tongue technology was firstly applied to assess the bitterness of AH. Based on the six taste factors, the PCA score 3D plot (Figure 2) exhibited a clear grouping trend (R^2^X, 0.912; Q^2^, 0.763) among the three different commercial classifications of samples: leaves, stems, and mixtures of both. The results implied that electronic tongue had the ability to distinguish the bitterness among different commercial classifications. Six compounds (peaks 2, 3, 4, 6, 7, and 8) with positive correlations to bitterness were discovered by Spearman correlation analysis. Furthermore, the peaks 2, 6, 7, and 8 were identified as andrographolide, neoandrographolide, 14-deoxyandrographolide, and dehydroandrographolide, respectively. In summary, detecting bitterness via electronic tongue technology could evaluate the quality of AH samples rapidly and efficiently.

## Figures and Tables

**Table 1:** Taste intensities of each taste factor in all AH samples (mean ± RSD, *n* = 3) and the Radar curve area.

Samples	B-Bitterness2	Aftertaste-B	Aftertaste-A	H-Bitterness	Bitterness	Astringency	Radar Curve Area
L1	6.83 ± 0.01	0.55 ± 0.05	0.19 ± 0.03	0.13 ± 0.04	3.86 ± 0.03	2.12 ± 0.05	11.713
L2	7.94 ± 0.05	0.51 ± 0.03	0.21 ± 0.08	0.11 ± 0.05	3.82 ± 0.03	2.25 ± 0.04	13.449
L3	5.90 ± 0.04	0.47 ± 0.05	0.10 ± 0.06	0.04 ± 0.07	3.16 ± 0.05	1.66 ± 0.03	7.790
L4	6.57 ± 0.02	0.49 ± 0.02	0.15 ± 0.07	0.09 ± 0.06	3.39 ± 0.06	1.94 ± 0.01	9.931
L5	8.53 ± 0.06	0.41 ± 0.05	0.19 ± 0.06	0.24 ± 0.04	3.28 ± 0.06	1.99 ± 0.03	12.085
ST1	2.79 ± 0.03	0.29 ± 0.05	0.07 ± 0.07	0.14 ± 0.07	2.06 ± 0.05	1.36 ± 0.06	3.344
ST2	1.30 ± 0.04	0.30 ± 0.05	0.10 ± 0.06	0.08 ± 0.08	2.39 ± 0.04	1.62 ± 0.05	2.857
ST3	6.07 ± 0.05	0.34 ± 0.03	0.09 ± 0.00	0.21 ± 0.07	2.14 ± 0.07	1.21 ± 0.09	5.411
ST4	7.17 ± 0.01	0.42 ± 0.01	0.11 ± 0.09	0.25 ± 0.06	2.30 ± 0.06	1.23 ± 0.09	6.629
ST5	5.48 ± 0.05	0.47 ± 0.05	0.09 ± 0.06	0.17 ± 0.06	2.38 ± 0.05	1.15 ± 0.04	5.229
ST6	5.99 ± 0.03	0.30 ± 0.04	0.06 ± 0.05	0.18 ± 0.06	2.13 ± 0.06	1.14 ± 0.05	4.965
ST7	8.51 ± 0.08	0.46 ± 0.07	0.16 ± 0.06	0.24 ± 0.09	2.88 ± 0.04	1.51 ± 0.05	9.490
ST8	5.65 ± 0.03	0.25 ± 0.02	0.08 ± 0.08	0.17 ± 0.07	2.03 ± 0.03	1.26 ± 0.02	4.966
ST9	5.83 ± 0.08	0.28 ± 0.08	0.10 ± 0.06	0.18 ± 0.03	2.05 ± 0.03	1.29 ± 0.02	5.288
ST10	7.28 ± 0.01	0.52 ± 0.01	0.12 ± 0.05	0.19 ± 0.03	2.53 ± 0.02	1.26 ± 0.12	7.237
ST11	7.21 ± 0.02	0.30 ± 0.05	0.08 ± 0.07	0.16 ± 0.06	1.90 ± 0.03	1.07 ± 0.04	5.305
ST12	3.87 ± 0.08	0.41 ± 0.05	0.05 ± 0.12	0.19 ± 0.03	2.41 ± 0.01	1.23 ± 0.05	4.243
ST13	7.92 ± 0.01	0.43 ± 0.04	0.12 ± 0.05	0.24 ± 0.07	3.23 ± 0.03	1.91 ± 0.01	11.067
S1	0.93 ± 0.05	0.20 ± 0.05	0.05 ± 0.10	0.02 ± 0.00	2.07 ± 0.05	1.40 ± 0.06	1.922
S2	1.87 ± 0.04	0.18 ± 0.06	0.02 ± 0.03	0.11 ± 0.05	1.58 ± 0.06	1.03 ± 0.08	1.762
S3	2.08 ± 0.07	0.15 ± 0.07	0.01 ± 0.11	0.08 ± 0.07	1.38 ± 0.03	0.99 ± 0.08	1.667
S4	1.96 ± 0.04	0.24 ± 0.08	0.06 ± 0.06	0.09 ± 0.06	1.90 ± 0.02	1.14 ± 0.07	2.192
S5	3.03 ± 0.03	0.24 ± 0.09	0.04 ± 0.13	0.11 ± 0.05	1.42 ± 0.08	0.86 ± 0.06	2.046
S6	1.82 ± 0.08	0.23 ± 0.07	0.05 ± 0.11	0.07 ± 0.09	1.56 ± 0.04	0.81 ± 0.05	1.421
S7	4.09 ± 0.01	0.22 ± 0.05	0.03 ± 0.10	0.13 ± 0.08	1.38 ± 0.03	0.86 ± 0.05	2.509
S8	1.00 ± 0.07	0.41 ± 0.05	0.07 ± 0.09	0.02 ± 0.00	2.63 ± 0.03	1.37 ± 0.03	2.367
S9	3.21 ± 0.02	0.29 ± 0.08	0.09 ± 0.07	0.17 ± 0.09	1.76 ± 0.03	1.10 ± 0.04	2.932
S10	1.31 ± 0.02	0.30 ± 0.03	0.05 ± 0.11	0.11 ± 0.09	2.03 ± 0.02	1.23 ± 0.03	2.055
S11	1.43 ± 0.09	0.22 ± 0.00	0.02 ± 0.07	0.12 ± 0.05	1.60 ± 0.03	0.99 ± 0.02	1.521
S12	1.84 ± 0.03	0.21 ± 0.07	0.01 ± 0.09	0.13 ± 0.04	1.71 ± 0.04	1.14 ± 0.01	2.017

L: represents leaf samples; ST: represents the mixed of stem and leaf samples; S: represents stem samples, *n* = 3 means one prepared sample was repeatedly tested 3 times according to the procedure.

**Table 2:** Correlation Coefficients between bitter (radar curve area) and the common peaks in HPLC fingerprint.

Common Peaks	Radar Curve Area	Common Peaks	Radar Curve Area
1	*ρ* = −0.125 (*p* = 0.510)	6	*ρ* = 0.854 (*p* < 0.01)
2	*ρ* = 0.725 (*p* < 0.01)	7	*ρ* = 0.890 (*p* < 0.01)
3	*ρ* = 0.729 (*p* < 0.01)	8	*ρ* = 0.691 (*p* < 0.01)
4	*ρ* = 0.629 (*p* < 0.01)	9	*ρ*=−0.826 (*p* < 0.01)
5	*ρ* = −0.014 (*p* = 0.942)		

**Table 3:** AH collection information.

Samples	Origin	Collection Parts	Samples	Origin	Collection Parts
L1	Anhui	Leaf	SL11	Guangdong	Stem/Leaf
L2	Anhui	Leaf	SL12	Anhui	Stem/Leaf
L3	Anhui	Leaf	SL13	Anhui	Stem/Leaf
L4	Anhui	Leaf	S1	Jiangxi	Stem
L5	Anhui	Leaf	S2	Anhui	Stem
SL1	Jiangxi	Stem/Leaf	S3	Guangxi	Stem
SL2	Jiangxi	Stem/Leaf	S4	Guangxi	Stem
SL3	Guangxi	Stem/Leaf	S5	Guangxi	Stem
SL4	Fujian	Stem/Leaf	S6	Guangxi	Stem
SL5	Guangxi	Stem/Leaf	S7	Guangdong	Stem
SL6	Guangxi	Stem/Leaf	S8	Guangdong	Stem
SL7	Guangxi	Stem/Leaf	S9	Sichuan	Stem
SL8	Guangdong	Stem/Leaf	S10	Sichuan	Stem
SL9	Guangdong	Stem/Leaf	S11	Jiangsu	Stem
SL10	Guangdong	Stem/Leaf	S12	Jiangsu	Stem

**Table 4:** Taste information represented by four taste sensors.

Sensor Probes	Taste Information	
	Initial Value	Aftertaste Value
C00	Bitterness	Aftertaste of anionic bitterness (aftertaste-B)
BT0	-	Aftertaste of cationic bitterness (H-bitterness)
AN0	-	Aftertaste of mineral bitterness (B-bitterness2)
AE1	Astringency	Aftertaste of astringency (aftertaste-A)

“-“ indicates no relative value.
